# Hyperbranched Liquid Crystals Modified with Sisal Cellulose Fibers for Reinforcement of Epoxy Composites

**DOI:** 10.3390/polym10091024

**Published:** 2018-09-14

**Authors:** Qiyun Luo, Yuqi Li, Li Ren, Xu Xu, Shaorong Lu

**Affiliations:** Key Laboratory of New Processing Technology for Nonferrous Metals and Materials, Ministry of Education, School of Material Science and Engineering, Guilin University of Technology, Guilin 541004, China; luoqiy2011@163.com (Q.L.); renli20011@163.com (L.R.)

**Keywords:** sisal cellulose fibers, epoxy resin, composites, liquid crystals, hyperbranched

## Abstract

Well-defined functionalized sisal cellulose fibers (SCFs) grafted on hyperbranched liquid crystals (HLP) were synthesized to improve the compatibility between SCFs and epoxy resin (EP). The influence of SCFs-HLP on the mechanical and thermal properties of SCFs-HLP/EP composites was studied. The results show that the mechanical properties of SCFs-HLP/EP composites were enhanced distinctly. Particularly, compared with EP, impact strength, tensile strength, and flexural strength of composites with 4.0 wt % SCFs-HLP were 38.3 KJ·m^−2^, 86.2 MPa, and 150.7 MPa, increasing by 118.7%, 55.6%, and 89.6%, respectively. As well, the glass transition temperature of the composite material increased by 25 °C. It is hope that this work will inform ongoing efforts to exploit more efficient methods to overcome the poor natural fiber/polymer adhesion in the interface region.

## 1. Introduction

Natural fibers (NFs) are one of the most abundant and renewable natural materials that reinforce polymer matrix composites to replace synthetic fibers because of the advantages, including cost effectiveness, biodegradability, light weight, and renewability [1,2,3]. Natural fibers have different origins such as sisal hemp, cotton, wood, bamboo, cereal straw, etc. [4,5,6,7]. Every year approximately 10-10 to 10-11 tons of NFs are produced by photosynthesis [8]. Natural fiber waste such as sorghum stalks, cereal crops, and corn stalks not only waste resources but also pollute the environment. Therefore, the study of NFs as reinforcement in thermoplastic composites has rapidly expanded in recent years, and there is tremendous potential for future growth [9,10].

However, NFs as reinforcement in thermoplastic composites have the significant drawback that the poor adhesion between the fibers and polymer matrices is generally insufficient, which hinders the application of NFs in composites [11,12,13,14]. To overcome this drawback, surface modification of NFs can be carried out. The modification of NFs by physical or chemical methods can effectively improve interface compatibility between NFs and epoxy matrices to improve the comprehensive properties of the composite [15,16]. The chemical methods will introduce new moieties that can effectively combine with epoxy matrix, such silanization, acetylation, alkalization, etc. [17]. Additionally, Carlmark et al. [18] investigated the effect of ring-opening polymerization on the mechanical properties of microfibrillated cellulose-reinforced polyester composites.

In the last decade, hyperbranched polymers have been used to modified the surface of NFs, the terminal groups of NFs can be reacted with functional groups of epoxy resins (EPs) to improve the compatibility between NFs and epoxy resin [19]. Furthermore, hyperbranched polymer contains three dimensional space structures in favor of the reinforcement of epoxy resin [20,21,22]. 

The aim of this study is to focus on the modification of the sisal cellulose fibers (SCFs) with function end-group hyperbranched liquid crystals (HLPs) for preparation of SCFs-HLP/EP composites. The HLPs were introduced to improve the compatibility between the SCFs and EP, with respect to improve the mechanical and thermal properties of SCFs-HLP/EP composites. A vital purpose of this study is the use of renewable resources to improve the EP matrix composites, instead of the application of synthetic fibers in EP matrix composites.

## 2. Experiment Section

### 2.1. Materials

The sisal fibers used as reinforcement were obtained from the Guangxi Sisal Company, Nanning, China. Their parameters consisted of the density, diameter, and length which were 1.30 g/cm^3^, 0.1 mm, and about 15 cm, respectively. The epoxy matrices were supplied by Yueyang Chemical Plant, Yueyang, China. Their parameters were diglycidylether of bisphenol A (DGEBA, E-44, epoxy value = 0.44), and 4,4-diaminodiphenylsulfone (DDS) was used as a curing agent, which was purchased from and the glycidol was also purchased from Aladdin Chemistry Co., Ltd., Beijing, China. Drugs such as tetrahydrofuran, 1,4-dioxane, toluene-2,4-diisocyanate (TDI), and *N*-methy-2-pyrrolidone (NMP) were provided by Sinopharm Chemical Reagent Co. Ltd., Beijing, China and were used with further purification. 6-Chloro-1-hexanol was purchased from Chengdu Gracia Chemical Technology Co. Ltd, Chengdu, China. Other chemicals, such as methyl alcohol, ethyl alcohol, sodium hydroxide (NaOH), acetic acid (CH_3_COOH), sodium sulfate (Na_2_S·10H_2_O), and sodium chlorite (NaClO_2_) were obtained from Guangzhou Jinhua du Chemical Reagent Co. Ltd., Jinhua, China and were used without further purification.

### 2.2. Preparation of Sisal Cellulose Fibers (SCFs)

Prior to extracting fibers, the sisal were washed with distilled water several times, then vacuum dried under 60 °C for 24 h. 10 g the washed fibers were put into a 200 mL reaction kettle to which a mixture solution of 4 g NaOH, 4 g Na_2_S·10H_2_O, and 100 mL distilled water were added, and then the mixture was heated to 170 °C for 2 h. After reaction, the products were filtered and washed several times with distilled water. Then the fibers were submerged in a solution of 3.35 g NaClO_2_, 325 mL water, and 2.5 mL CH_3_COOH for 2 h at 80 °C. The products were washed several times with distilled water and dried in a vacuum oven at 75 °C until constant weight.

### 2.3. The SCF-Grafted Hyperbranched Polyglycerol on the Surface (SCFs-HPG)

**Scheme 1:**
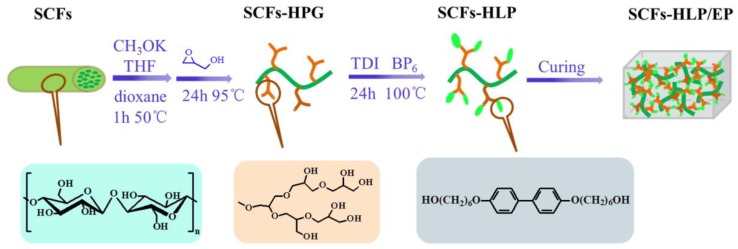
The schematic of the preparation of hyperbranched liquid crystals modified sisal cellulose fibers/epoxy resin (SCFs-HLP/EP) composites.

Functionalization of SCFs was carried out through ring-opening multibranching polymerization of glycidol (Scheme 1). Firstly, 0.08 g SCFs, 5.00 mL tetrahydrofuran, and 20.0 uL CH_3_OK were put in a three-necked flask with a reflux condenser and magnetic stirring. The mixture solution was stirred at 50 °C for 1 h, then 10 mL dioxane was added slowly. Subsequently, 1.8 mL glycidol was poured into the mixture solution and heated to 100 °C for 30 h, then 1.8 mL glycidol was added slowly for 24 h in order to hinder self-ring-opening polymerization of glycidol. After cooling to room temperature, the products were purified by washing several times with methanol and then dried.

### 2.4. Preparation of 4,4-di (β-hydroxy ethoxy) Biphenyl (BP6)

The preparation of BP6 was as followed: 2.24 g KOH and 0.3 g KI were dissolved in 90 mL anhydrous ethanol, then 2.2 g of 4,4-biphenol was added into the mixture and stirred. After the dissolution of the 4,4-biphenol, 10.88 g 6-chloro-1-hexanol was poured into the mixture solution slowly and heated to 75 °C for 2 h. Subsequently, the temperature was cooled to ambient temperature and the products were washed several times with distilled water. Lastly, the products were purified by recrystallization, after which the products were dried in a vacuum oven at 60 °C for 24 h.

### 2.5. Preparation of SCFs-HLP

As shown in Scheme 1, 0.1 g SCFs-HLP and 20 mL NMP were put into a 100 mL three-neck flask. The reaction was kept at 100 °C under a nitrogen atmosphere. Subsequently, 0.285 g of TDI was added and stirred for 5 h. Then the reaction mixture was put in 0.35 g of BP6 and stirred for 24 h. The resulting products were obtained by filtration and further purification.

### 2.6. Preparation of the SCFs-HLP/EP Composites

Took the synthesis of SCFs-HLP/EP composites with 1.0 wt % SCFs-HLP as an example: 26 g EP was weighed and heated to have a good liquidity. In the meantime, 0.26 g of SCFs-HLPs were dispersed in 10 mL acetone through ultrasonication for 30 min. Then the SCFs-HLP/acetone suspension was poured into the preheated EP. The mixture solution was stirred until the acetone nearly volatilized. Subsequently, 0.78 g DDS was added and stirred until entirely dissolved. The mixture was degassed until there were no bubble formations in the mixture solution. Finally, the mixture solution was poured into a preheated stainless steel mold and degassed in a vacuum drying oven at 120 °C for 30 min. Then the mold was thrown in a drying oven had been set at 140 °C for 2 h, 160 °C for 2 h, and 180 °C for 2 h in advance. The experimental steps are shown in Scheme 1.

### 2.7. Characterizations

Thermogravimetric analysis (TGA) was examined with a NETZSCH STA 449C, (NETZSCH instrument, Bavaria, Germany). The tests were performed under a nitrogen atmosphere with a constant heating rate of 10 °C min^−1^. Dynamic mechanical analysis (DMA) was performed with a DMA Q800 (TA instruments, New Castle, DE, USA) using a frequency of 1.0 Hz at a heating rate of 3 °C cm^−1^. Fourier transformed infrared spectroscopy (FTIR) was recorded on a Perkin–Elmer 1710 spectrophotometer (PerkinElmer instruments, Waltham, MA, USA) in a range from 4000 to 500 cm^−1^. The morphology of samples was studied by scanning electron microscopy (FE-SEM, JEOL JSM-6701F, Tokyo, Japan). X-ray photoelectron spectroscopy (XPS) was carried out by a VG-microtech ESCA2000 spectrometer (VG Microtech Inc. Eugene, OR, USA) in order to analyze qualitatively the functionalization of SCFs-HLP. X-ray diffraction (XRD) patterns were recorded using an X-ray diffractometer (X’Pert PRO, Panalytical B.V., Almelo, Holland) the samples were scanned from 2θ values of 5 to 40°. Optical structure was performed by a hot-stage polarizing microscopy. The mechanical properties of the specimen were characterized by impact, tensile, and fexural measurements. The impact test was carried out with a tester of type XJJ-5 in terms of the National Standard of China (GB1043-79). The tensile test was examined by a tensile tester of type RGT-5 at a rate of 2 mm/min. The flexural test was studied by a tester of type WDW-20 using a three-point bending mode of the universal testing machine.

## 3. Results and Discussion

### 3.1. The Characterization of SCFs-HLP 

**Figure 1:**
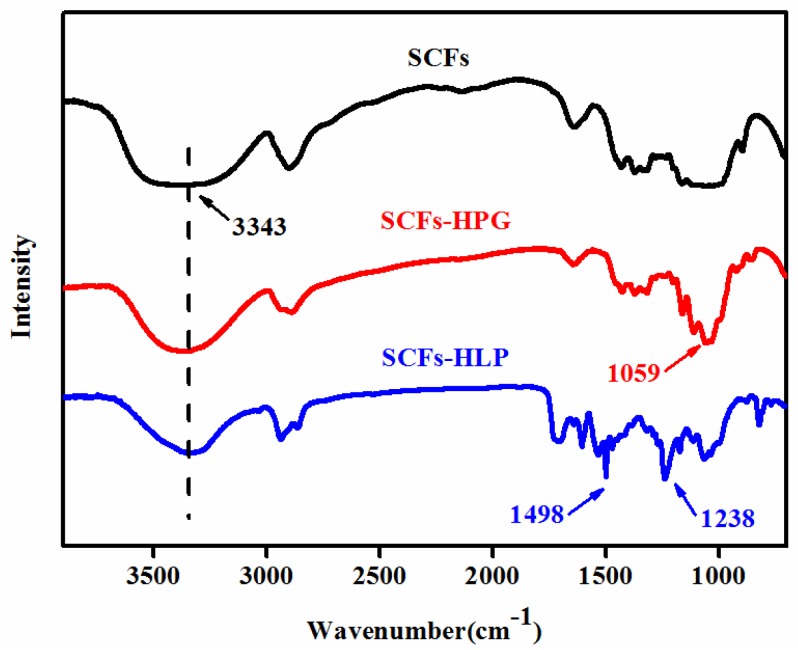
FTIR spectra of sisal cellulose fibers (SCFs), Hyperbranched Polyglycerol modified SCFs (SCFs-HPG) and Hyperbranched liquid crystals modified SCFs (SCFs-HLP).

The FTIR was used to study the structure of SCFs and its ramification. Figure 1 shows the FTIR of SCFs and the surface modification of SCFs. From Figure 1, we can see that the spectrum of SCFs and that of the SCFs-HPG have a similar trend. However, the absorption peak of SCFs-HLP shows the feature peak of C-O-C stretching at 1059 cm^−1^. The ring-opening reaction of glycidol generated hyperbranched polyglycerol (HPG), which included C-O-C bonds. This can be explained by the HPG grafted onto the SCFs successfully. Moreover, the absorption peak of the SCFs-HLP shows that the feature peak of the benzene ring was at 1498 cm^−1^ (aromatic, C=C stretching) and 1238cm^−1^ (aromatic, C-H bending), which indicates that the HLP grafted on the SCFs successfully.

**Figure 2:**
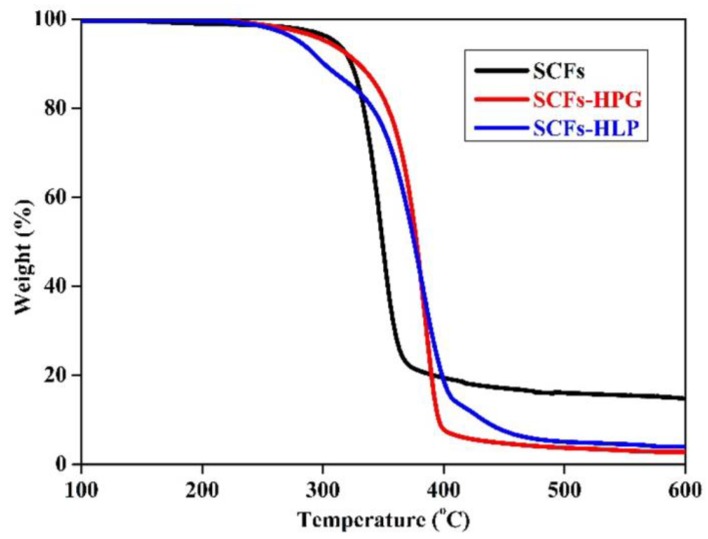
Thermogravimetric analysis (TGA) thermograms of SCFs, SCFs-HPG, and SCFs-HLP.

The thermal degradation process of SCFs and its ramifications were studied by TGA, which was used to research the thermal stability of SCFs and its ramification. As shown in Figure 2, the initiate degradation of SCFs-HPG and SCFs-HLP shifted to a lower initiate degradation temperature compared with that of SCFs. Because of the degradation of HPG and HLP. Moreover, the weight loss of SCFs-HPG and SCFs-HLP was higher than that of SCFs because the HPG and HLP was grafted on SCFs, because of the HPG and HLP carbonized at lower temperatures. To summarize what has been mentioned above, the HPG and HLP was grafted onto the surface of SCFs successfully.

**Figure 3:**
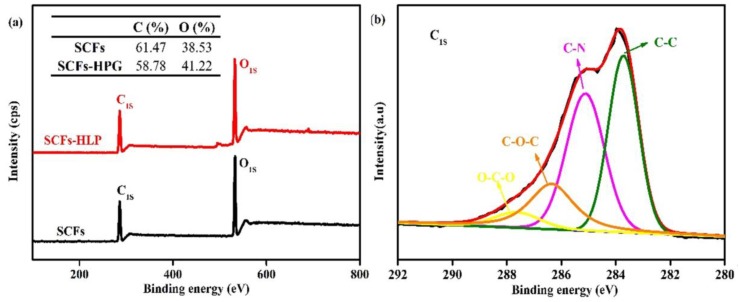
The X-ray photoelectron spectroscopy (XPS) spectrums of SCFs, SCFs-HPG, and SCFs-HLP: (**a**) the low-resolution survey scans of C1S and O1S for SCFs and SCFs-HPG; and (**b**) the high-resolution survey spectrums of C1S for SCFs-HLP.

As shown in Figure 3, XPS were carried out to analyses for SCFs, SCFs-HPG, and SCFs-HLP. In Figure 3a, SCFs and SCFs-HPG exhibited the same elements, including carbon and oxygen, except for hydrogen, which cannot be tested by XPS. However, The O/C ratios of SCFs and SCFs-HPG were 0.62 and 0.70, respectively. The change of O/C ratios verified that the HPG grafted onto the SCFs successfully, because the O/C ratios of the HPG was higher than that of SCFs. In Figure 3b, the C1S peak was decomposed into four peaks, which is contributed to C-C, C-N, C-O-C, and O-C-O, respectively. The existence of C-N indicated that the SCFs-HLP was synthesized successfully.

**Figure 4:**
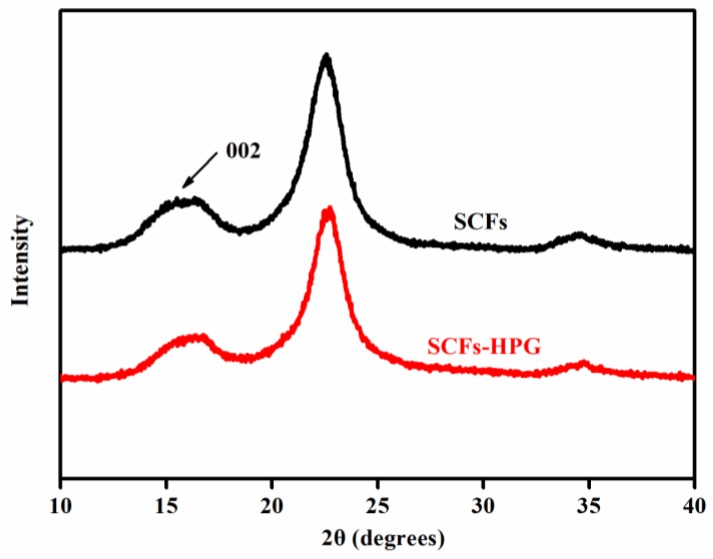
X-ray diffraction (XRD) pattern of SCFs and SCFs-HPG.

In Figure 4, XRD patterns of SCFs and SCFs-HPG show similar trends, which indicate that the HPG grafted onto SCFs have limited influence on the structure of SCFs. The 002 peak corresponds to characteristic peaks for crystallinity in SCFs. It can be seen that the crystallinity of SCFs-HPG was lower than that of SCFs. This implies that the reaction of SCFs with functionalized HPG occurred in the amorphous regions and penetrated into the crystalline regions of SCFs [23].

**Figure 5:**
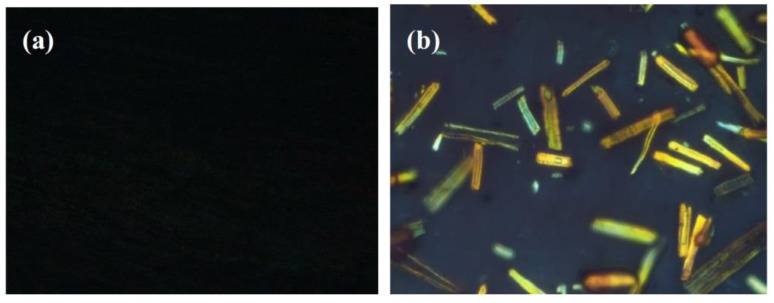
Polarizing microscope (POM) of SCFs-HLP at room temperature (**a**) and at melting point (**b**).

In Figure 5, the optical structure of SCFs-HLP was studied with a polarizing microscope (POM). As shown in Figure 5a, the structure of SCFs-HLP at room temperature cannot be seen under polarized light. However, in Figure 5b, as the temperature was raised to the melting point of the HLP, the HLP began to melt and the liquid crystal birefringence appeared, which indicates that the surface of SCFs was grafted with the HLP successfully. In Figure 5b, the morphologies of the HLP-SCFs exhibited characteristic optical textures of mesogenic under polarized light.

### 3.2. Thermal Properties of the Composites

**Figure 6:**
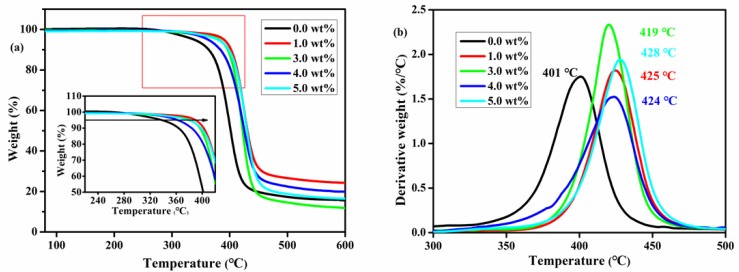
TGA and Differential thermal gravity (DTG) curves of neat epoxy and its composites.

The TGA and Differential thermal gravity (DTG) curves of neat epoxy and its composites are shown in Figure 6. They were measured using thermogravimetric analysis at a heating rate of 10 °C/min under nitrogen conditions. As shown in Figure 6a, the neat epoxy and its composites exhibited similar degradation curve, which can be interpreted that by the changed degradation mechanism of SCFs-HLP/EP after added the SCFs-HLP. The composites have a higher decomposition temperatures (*T*_d_) at 5% weight loss compared with EP. In other words, the thermal stability of composites improved effectively with increasing SCFs-HLP fillers. Furthermore, the maximum degradation temperature (*T_max_*) of neat epoxy and its composites is shown in Figure 6b. It can be observed that the *T_max_* of composites enhanced obviously with increasing SCFs-HLP fillers. The *T_max_* increased from 401 to 428 °C by increasing the SCFs-HLP fillers, which increased 27 °C compare with the EP. This is attributed to the three-dimensional architecture of SCFs-HLP, which can effectively hinder the movement of EP molecular chains [24]. The SCFs-HLP’s own large numbers of end groups interacted well with the end groups of EP [25], which also can prevent the movement of EP molecular chains.

**Figure 7:**
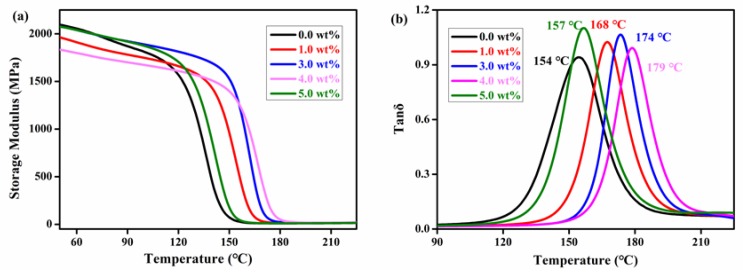
Dynamic mechanical properties of EP and its composites.

The storage modulus and tanδ of EP and its composites are shown in the Figure 7. The change of the storage modulus (E′) with increasing SCFs-HLP contents are shown in Figure 7a. From Figure 7a, we can see that the curve of the samples have a similar tendency, which shows a tendency to decrease with the increase in temperature. This is due to the movement of the polymer segments leading to energy dissipation. In addition, the storage modulus of composites with increasing SCFs-HLP fillers was higher than that of EP after glass transition. Due to the movement of epoxy resin segments decreases as the SCFs-HLP content increases. This can also be testified by Figure 7b. The peak of tanδ is defined as the glass transition temperature (*T*g) in which the polymer transforms the glass state to the high elastic state. As shown in Figure 7b, the Tg of SCFs-HLP/EP composites increase with the increasing of SCFs-HLP contents, especially when the *T*g of SCFs-HLP/EP composites with 4.0 wt % SCFs-HLP was 179 °C, which increased to 25 °C compared to that of EP (154 °C). There are three factors to explain these results. Firstly, the movement of the segments is hindered by the interaction of SCFs-HLP and EP [26,27]. In another aspect, the special three-dimensional network structure of hyperbranched polymer SCFs-HLP can effectively prevent the movement of the chain segment [28,29]. Furthermore, the rigid benzene ring structure of SCFs-HLP like nails embed in the EP to prevent the movement of the chain segment [30]. In other words, the crosslinking density of SCFs-HLP/EP composites increase with the addition of SCFs-HLP, which reduced the movement of the segments and increased the *T*g of SCFs-HLP/EP composites [31,32]. However, when the SCFs-HLP contents were over 5.0 wt %, the *T*g of SCFs-HLP/EP composites decrease significantly. It can be attributed to the agglomeration of SCFs-HLP. The interaction between the SCFs-HLP and the matrix can be reduced by the agglomeration of SCFs-HLP, which lead to the *T*g decrease. Moreover, the sharpness of the peak was an important indicator to assess the compatibility of composites. If the composites have an excellent compatibility, the peak of tanδ displays a sharp peak; on the contrary, the peak observed a relatively broad peak.

### 3.3. Mechanical Properties of the Composites

**Figure 8:**
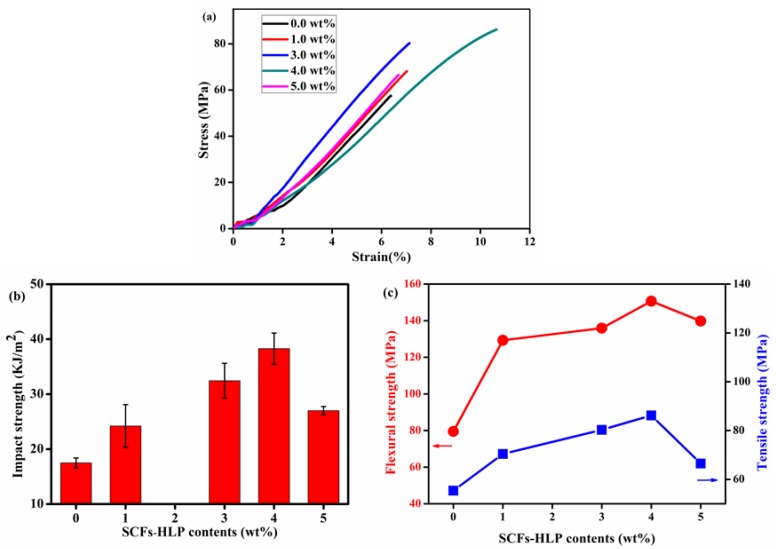
(**a**) Stress-strain curves of EP and SCFs-HLP/EP composites; (**b)** impact strength of EP and SCFs-HLP/EP composites; and (**c**) flexural strength and tensile strength of EP and SCFs-HLP/EP composites.

The stress-strain curves of epoxy resin modified with 0–5 wt % SCFs-HLP content are presented in Figure 8a. The result show that the stress-strain curves of neat epoxy ehxibited a linear growth at the breaking point, due to the neat epoxy was brittle and fractured. However, the composites with 4.0 wt % SCFs-HLP showed higher elongation at break, which explains how the addition of SCFs-HLP can improve the plastic deformation of EP. Impact strength is one of indicators which evaluates the mechanical properties of composites. The impact strength of neat epoxy and its composites with different SCFs-HLP contents is shown in Figure 8b. Compared with neat epoxy, the impact strength of composite contents improved with different SCFs-HLP. The peak value of impact strength was obtained at a loading with 4.0 wt % of SCFs-HLP contents reaching 38.28 KJ·m^−2^, which increased 118.7% compared with neat epoxy. The tensile strength and flexural strength of neat epoxy and its composites with different SCFs-HLP contents are shown in Figure 8c. The tensile strength and flexural strength of SCFs-HLP/EP composites exhibited a similar trend. The tensile strength and flexural strength of SCFs-HLP/EP composites obtained peak values of 86.2 MPa, 150.7 MPa with 4.0 wt % SCFs-HLP contents, respectively, which increased by 55.6% and 89.6% compared with the EP. Table 1 shows the mechanical properties of neat epoxy and its composites, which include the impact strength, tensile strength, and flexural strength. The results show the similar trend of increase with the adding of SCFs-HLP contents, reducing the adding trend after. This is accounted for by the enhancement of interfacial interactions between SCFs-HLP and EP. On the other hand, the SCFs-HLP, as a dendritic polymer, has a three-dimensional architecture which can effectively enhance the toughness of EP, and then the SCFs-HLP has abundant functional end groups which can interact with the functional groups of EP, which can enhance the interfacial interactions between SCFs-HLP and EP. In addition, the SCFs-HLP contain rigid benzene structures, which can be used to prevent the development of the microcrack and the orientation of the mesogenic units. However, when the content of SCFs-HLP exceeds 4.0 wt %, the mechanical properties of SCFs-HLP/EP composites decreased. It can be attributed to the poor dispersion of SCFs-HLP, which lead to the increasing of defects between SCFs-HLP and EP [33].

**Figure 9:**
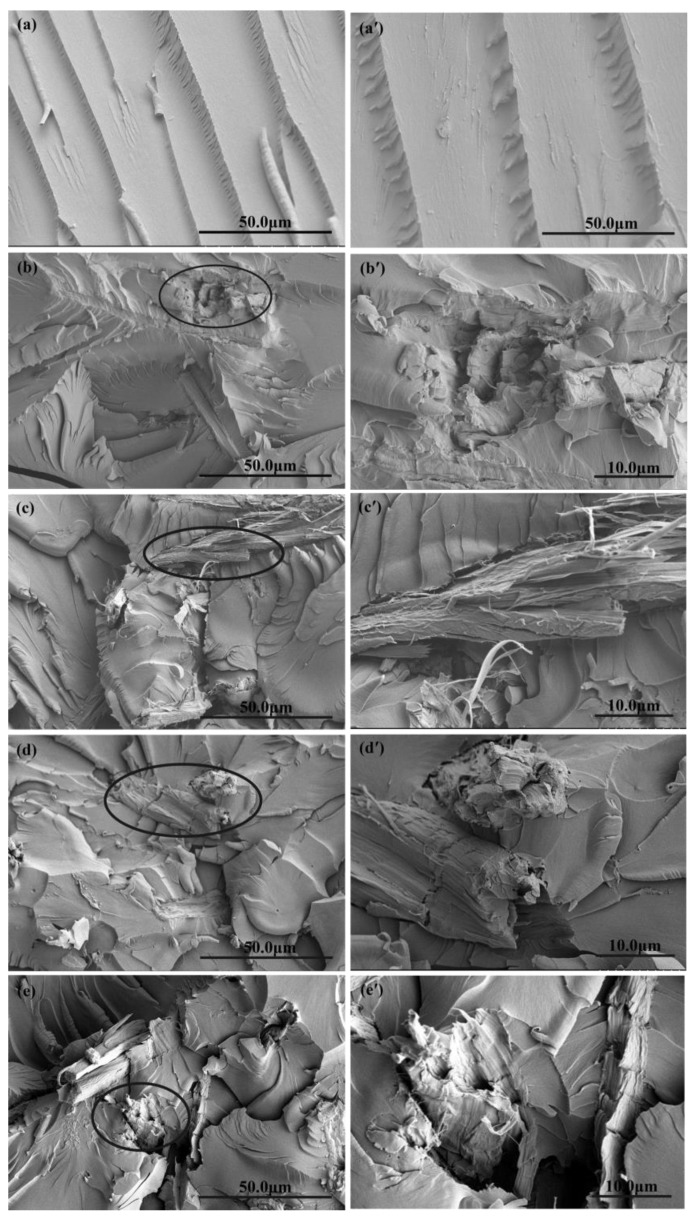
SEM images of fractured surface: (**a**,**a′**) neat epoxy, and SCFs-HLP/EP composites: (**b**,**b′**) 1.0 wt %, (**c**,**c′**) 3.0 wt %, (**d**,**d′**) 4.0 wt %, and (**e**,**e′**) 5.0 wt %.

As shown in Figure 9, the fracture surface of EP and its composites were obtained by SEM. In Figure 9a,a′, it was found that the fracture surface of neat epoxy was very smooth and had poor resistance to crack propagation, which can be verified by the typical brittle facture of EP. The fracture surface of SCFs-HLP/EP composites with different SCFs-HLP content are shown in Figure 9b,b′,e,e′. Compared with neat epoxy, the fracture surface of SCFs-HLP/EP composites displayed a rougher surface, and saw good compatibility between SCFs and EP. The SCFs can reduce fracture energy and hinder crack propagation, which indicates the change in impact strength of SCFs-HLP/EP composites in Figure 8b. All these observations verified that surface modification of SCFs can improve the interfacial adhesion between SCFs and the epoxy matrix.

## 4. Conclusions

In this work, novel functionalized sisal cellulose fibers (SCFs) grafted with hyperbranched liquid crystals (HLP) were carried out to improve the compatibility between the SCFs and an epoxy resin (EP) matrix. The mechanical and thermal properties of the composites with SCFs-HLP were studied by impact strength, tensile strength, flexural strength, TG, and and DMA. The result shows that the addition of MCFs–HLP filler to EP matrices improves the mechanical and thermal properties effectively. Especially, at 4.0 wt % SCFs-HLP, the impact strength, tensile strength, flexural strength of the composites were 38.3 KJ·m^−2^, 86.2 MPa, and 150.7 MPa, respectively, which increased by 118.9%, 55.6%, and 89.6% compared with that of EP. Furthermore, the glass transition temperature increased by 25 °C. In conclusion, the surface modification of SCFs with HLP should be a promising method to improve the compatibility between the SCFs and EP matrices.

## Figures and Tables

**Table 1:** Mechanical properties of EP and its composites.

SCFs-HLP (wt %)	Impact Strength (KJ m^−2^)	Tensile Strength (MPa)	Flexural Strength (MPa)
0.0	17.5 ± 0.9	55.4 ± 1.3	79.5 ± 2.5
1.0	24.2 ± 3.8	70.4 ± 5.5	129.3 ± 4.2
3.0	32.5 ± 3.2	80.3 ± 6.1	135.9 ± 4.8
4.0	38.3 ± 2.8	86.2 ± 1.2	150.7 ± 4.2
5.0	27.0 ± 0.7	66.5 ± 4.3	139.8 ± 6.4
